# Increased Autophagy of Rice Can Increase Yield and Nitrogen Use Efficiency (NUE)

**DOI:** 10.3389/fpls.2019.00584

**Published:** 2019-05-07

**Authors:** Jinlei Yu, Xiaoxi Zhen, Xin Li, Nan Li, Fan Xu

**Affiliations:** ^1^Key Laboratory of Northern Japonica Rice Genetics and Breeding, Ministry of Education and Liaoning Province – Key Laboratory of Northeast Rice Biology and Genetics and Breeding, Ministry of Agriculture, Rice Research Institute, Shenyang Agricultural University, Shenyang, China; ^2^Shenyang Product Quality Supervision and Inspection Institute, Shenyang, China

**Keywords:** autophagy, *OsATG8a*, nitrogen use efficiency, yield, rice

## Abstract

Autophagy (self-eating), a conserved pathway in eukaryotes, which is designed to handle cytoplasmic material in bulk and plays an important role in the remobilization of nutrient, such as nitrogen (N) under suboptimal nutrient conditions. Here, we identified a core component of an autophagy gene in rice (*Oryza sativa*), *OsATG8a*, with increased expression levels under N starvation conditions. Overexpression of *OsATG8a* significantly enhanced the level of autophagy and the number of effective tillers in the transgenic rice. In addition, the transgenic lines accumulated more N in grains than in the dry remains and the yield was significantly increased under normal N conditions. Further N allocation studies revealed that the nitrogen uptake efficiency (NUpE) and nitrogen use efficiency (NUE) significantly increased. Otherwise, under suboptimal N conditions, overexpression of *OsATG8a* did not seem to have any effect on yield and NUE, but NUpE was still improved significantly. Based on our findings, we consider *OsATG8a* to be a great candidate gene to increase NUE and yield.

## Introduction

Nitrogen (N) is one of the most important nutrient elements for plants and is required for growth and development. N is also a vital component of amino acids, proteins, nucleic acids, chlorophyll, and plant hormones (Kraiser et al., 2011). In plants, especially cereal crops, the yield and quality of the grain depend not only on the N absorbed prior to flowering, but also on the mobilization of reused N from mature leaves during senescence (Kichey et al., 2007; Chardon et al., 2012; Havé et al., 2017). The circulation process for organic nitrogen transfer from aging tissues to seeds in plants is an important determinant of productivity and yield, especially under nitrogen deficiency stress (Masclaux-Daubresse et al., 2010). Although the pathways of protein metabolism have not been thoroughly studied in senescing leaves, the following three important pathways are currently considered: plastid resident proteases, senescence-associated vacuoles (SAVs), and autophagy (Otegui et al., 2005; Ishida et al., 2008; Wada et al., 2009; Roberts et al., 2012).

Autophagy, a conserved vacuolar degradation pathway to remove cellular debris, appears to be active at a basal level in plant cells during all developmental stages, (Marshall and Vierstra, 2018). First, autophagy transfers the “cargo” (cytoplasmic components) to “degradation organs” (vacuoles in yeast and plants). Second, the cargo is degraded (Li and Vierstra, 2012). In response to stress or nutrient starvation, autophagy is enhanced to facilitate the degradation of increasing levels of toxic and damaged components and nutrients mobilized from this recycled cell material are then used to maintain cellular processes and in adaptation to stress (Yang and Bassham, 2015). Through studies on a variety of organisms, the mechanism of the autophagic machinery has emerged. The first AuTophaGy-related gene (*ATG* gene) was found in yeast (Miki and Yoshinori, 1993). Many orthologs of yeast *ATG* genes have been characterized in different species, including mammals and plants (Wang et al., 2015; Li et al., 2016; Zhai et al., 2016). To date, almost 40 ATG proteins have been identified as evolutionarily conserved among mammals, yeasts, and plant; these proteins form the core autophagic mechanism (Liu and Bassham, 2012).

A study of *Arabidopsis thaliana atg* mutants showed that autophagy controls the remobilization of N from leaves to seeds (Guiboileau et al., 2012). NUE decreased significantly to about 50% in the seeds of *atg* mutants compared to the WT under N-limited conditions (Guiboileau et al., 2013). Similar results were found in crops, such as in the rice *osatg7-1* mutant, where biomass and NUE during the vegetative growth stage were significantly reduced and the mutant was unable to mobilize N in aging leaves (Wada et al., 2015). Recent research further indicated that the metabolism and endogenous levels of gibberellins and cytokinin in anthers of the *osatg7* mutant were defective, which is the reason for defects in pollen maturation of autophagy-deficient plants (Kurusu et al., 2017). In maize (*Zea mays*), ^15^N pulse-chase analysis revealed that the amount of recycled N was reduced by two-fold in the *atg12* mutant compared with WT (Li F. et al., 2015). A facile multi-omics strategy in maize showed significant effects of autophagy on the formation of eukaryotic proteins and membranes under well-fed growth conditions and nutrient stress (Marshall and Vierstra, 2018). In addition, a study on the model plant *A. thaliana* also revealed a shorter life span, premature senescence of leaves, metabolome changes in cells, reallocated resources and protein accumulation, and impaired tolerance to biological and abiotic stress in *atg* mutants (Di et al., 2018).

Among the many plant ATG proteins, ATG8 is an important protein involved in formation of the autophagosome and recruitment of particular types of cargo (Kellner et al., 2016). The ATG8 protein is ubiquitin-like and conjugated to phosphatidylethanolamine (PE) on the autophagic membrane, which is located on the autophagosome membrane (Nakatogawa et al., 2007; Xie et al., 2008; Klionsky and Schulman, 2014). ATG8 is a central player in the autophagy process because it provides a docking site for ATG8 interaction motif (AIM)-containing autophagic receptors that selectively recruit cargo (Kellner et al., 2016). Seven *ATG8* isoforms (*OsATGa* to *OsATGg*) have been identified in the rice genome. *OsATG8a* through *OsATG8c* have high levels of identity with the deduced amino acid sequence and *OsATG8d* has similarities to *AtATG8i* (Chung et al., 2009; Xia et al., 2011); *OsATG8e*, however, has a gene locus but a lack of supporting expressed sequence tag (EST) data (Chung et al., 2009); *OsATG8f* only has a gene locus ID in RAP-DB but has no corresponding full-length cDNA; and *OsATG8i* has not been mapped to the rice genome (Xia et al., 2011). Several studies indicate that overexpression of *ATG8* homologs promotes plant growth and increases tolerance to N starvation in plants (Slavikova et al., 2008; Xia et al., 2012; Li W. et al., 2015, Li et al., 2016; Kellner et al., 2016; Wang et al., 2016).

In this article, some studies were done to identify the potential roles of *OsATG8a* (LOC_*Os07g32800*) on yield and N uptake and reuse in rice. The OsATG8a protein was predominantly localized to the cytoplasm, with autophagosome-like structures seen as fluorescence puncta. Compared to the control plants, the independent *35S-OsATG8a* transgenic lines had more effective tillering and produced more panicles and seeds, thus increasing yield. At the same time, autophagy levels were significantly enhanced in the transgenic lines under both N sufficient and deficient conditions. N allocation studies showed that overexpression of *OsATG8a* significantly increased N allocation to seeds and enhanced nitrogen uptake efficiency (NUpE) and nitrogen use efficiency (NUE). Based on these results, *OsATG8a* is an important candidate gene for synergistically enhanced N remobilization and better yield in rice.

## Materials and Methods

### Plant Materials and Growth Conditions

The japonica rice variety Shennong9816 (SN9816) was used in this study. For short term hydroponic culture, rice seedlings were grown in half Hoagland’s solution (with 3 mM KNO_3_ and 0.25 mM (NH_4_)_2_SO_4_, pH = 5.7) as previously described (Xia et al., 2012) in a growth chamber (28°C/25°C and 10 h light/14 h dark). To analyze the response of *OsATG8a* expression to N limitation in rice, rice seedlings were cultured with half Hoagland’s solution as sufficient N (NS, with 3.5 mM N) for 14 days, then transferred to the same NS (3.5 mM) solution as control; modified half Hoagland’s solution (with 0.6 mM KNO_3_ and 0.1 mM (NH_4_)_2_SO_4_, pH = 5.7) for low N (NL, with 0.8 mM N); and N-free solution for deficient N (ND, with 0 mM N); the lack of K^+^ was replaced with KCl in NL (0.8 mM) and ND (0 mM) solution. After 1 and 3 days of cultivation in NS (3.5 mM), NL (0.8 mM), or ND (0 mM) conditions described above, 10 rice seedlings (SN9816) of each N level were collected as a sample, and the leaves and roots were harvested separately.

### Total RNA Extraction and Gene Expression Analysis

Total RNA extraction was conducted using the Eastep Super Total RNA Extraction Kit (Promega) and first strand cDNA was synthesized with the PrimeScript RT Master Mix (TaKaRa). Real-time RT-PCR was performed as described previously (Xu et al., 2011) using SYBR Premix Ex TaqII (TaKaRa) on an Applied Biosystems 7500 Real Time PCR System. The following standard thermal profile was used for all PCRs: 95°C for 30 s, 40 cycles of 95°C for 5 s, and 60°C for 34 s. All reactions were done at least in triplicate. *OsActin1* was used as an internal control. The primers used for RT-PCR analysis are listed in Supplementary Table S1.

### Binary Vector Construction and Rice Transformation

The *OsATG8a* gene has a 360-bp coding sequence (CDS) and encodes 120 amino acids. The complete coding region of *OsATG8a* was amplified by PCR using SN9816 cDNA as a template and ligated into binary vector pCAMBIA1301, resulting in the *35S-OsATG8a* fusion gene. For the *Pro_OsATG8a_-GUS* construct, a 1405-bp promoter fragment of *OsATG8a* was amplified by PCR using SN9816 genomic DNA as templates and cloned into vector pCAMBIA1301. The *35S-eGFP-OsATG8a* (enhanced green fluorescent protein) fusion constructs were produced by inserting the complete coding region of *OsATG8a* into the pCAMBIA1300 vector. All constructs were confirmed by DNA sequencing. Sequences of all the primers used in cloning are listed in Supplemental Table S1. The plant expression vector was transformed into SN9816 by *Agrobacterium*-mediated transformation as described previously (Hiei et al., 1994). 14-day-old seedlings of *35S-OsATG8a* homozygous T_3_ lines and SN9816 grown hydroponically were sampled to confirm the transcript level of *OsATG8a.*

### Protein Isolation and Western Blot Analysis

Fourteen-day-old seedlings of transgenic lines and SN9816 cultured hydroponically in NS (3.5 mM) solution were transferred to NS (3.5 mM) or ND (0 mM) solution for 24 h to analyze the autophagic flux. Total protein extraction was conducted using the Minute^TM^ Total Protein Extraction Kit for plants (Invent Biotechnologies, Inc.), and total membrane protein extraction was conducted using the Minute^TM^ Membrane Protein Isolation Kit for plants (Invent Biotechnologies, Inc.). Protein concentration was determined with the BCA Protein Assay Kit (TaKaRa), and equal amount of protein was loaded in each lane. Immunoblotting was done as described previously (Chung et al., 2009; Xia et al., 2012). The obtained proteins were loaded into a 12% SDS-PAGE with 6 M urea gel. After electrophoresis separation, the proteins were transferred to a PVDF membrane for protein profile analysis or immunoblot analysis. Antibodies against *Arabidopsis* ATG8a and NBR1, α-Tubulin (Agrisera) were used as primary antibodies (1: 1000); goat anti-rabbit (IgG) (Sigma-Aldrich) was used as the secondary antibody (1: 10000). The reactions were detected using the Pro-light HRP Chemiluminescent Kit (TIANGEN). Quantification analysis of the protein band intensities from immunoblots was performed by software Image J. For total protein content of leaves and seeds analysis, proteins were extracted from 100 mg fresh weight of leaves at grain filling stage, 5 mg dry mass of seeds after harvested, and the same volume was loaded in each lane. Then, the total protein content was determined with the BCA Protein Assay Kit (TaKaRa) and Coomassie Brilliant Blue staining. Each experiment was repeated at least three times, and one representative result was shown.

### MDC Staining for Detecting Autophagosome Activity

Twenty one-day-old seedlings of transgenic lines and SN9816 cultured with NS (3.5 mM) solution were transferred to NS (3.5 mM) or ND (0 mM) liquid medium for 24 h, then the roots were excised for detecting autophagosome activity by monodansylcadaverine (MDC) staining. MDC staining was conducted as described (Wang et al., 2013), confocal images were acquired using an inverted Zeiss LSM 780 laser scanning microscope.

### Pot Trials of Rice

Rice plants were cultivated in pots under natural growth conditions from for three different years at Shenyang Agricultural University (Shenyang, China; Longitude: 123.34°E Latitude: 41.49°N), with each pot containing 14 kg of air-dried soil collected from an experimental farm at Shenyang Agricultural University. The growing season is from April to October every year. Field tests for rice using the T_3_ generation of *35S-OsATG8a* homozygous transgenic rice in the SN9816 background were carried out under natural growth conditions. There were three N levels in this experiment, N225 (225 kg ⋅ ha^–1^, 2.79 g ⋅ pot^–1^), N75 (75 kg ⋅ ha^–1^, 0.93 g ⋅ pot^–1^), and N0 (without N fertilizer). Urea was used as the only nitrogen source for N225 and N75 treatments and was applied three times during the whole growth period. For N225 or N75 treatment, the rice plants were applied with 81 kg ⋅ ha^–1^ or 26 kg ⋅ ha^–1^ urea as basal fertilizer, then 54 kg ⋅ ha^–1^ or 18 kg ⋅ ha^–1^ as tillering fertilizer, finally 90 kg ⋅ ha^–1^ or 30 kg ⋅ ha^–1^ as panicle fertilizer. Superphosphate and potassium chloride were used as phosphate and potassium fertilizers for each N level, superphosphate was supplied as basal fertilizer, and potassium chloride was supplied as basal fertilizer and twice as panicle fertilizer. There were three separate plots each with 3 N levels, each transgenic line or SN9816 planted ten pots for each N level per plot as replicates, and four plants were grown in a pot.

### GUS Histochemical Staining and Fluorometric Assay for GUS Activity

For temporal and spatial expression pattern analysis, tissues from the culm, leaf sheath, leaf blade, and developing seeds of *Pro_OsATG8a_-GUS* transgenic rice cultivated in pots under N225 conditions, and roots of 14-day-old transgenic seedlings cultured with NS (3.5 mM) solution were sampled for histochemical detection of GUS expression. For the N stress, 14-day-old seedlings of *Pro_OsATG8a_-GUS* transgenic rice cultured with NS (3.5 mM) solution were transferred to ND (0 mM) liquid medium for 24 h, then GUS histochemical staining was conducted as described previously (Xu et al., 2011), and the fluorimetric assay for GUS activity was performed as described previously (Jefferson, 1989).

### Subcellular Localization Assay

The *35S-eGFP-OsATG8a* fusion constructs were used to investigate the subcellular localization of OsATG8a. OsMCA1-RFP was used as a cell membrane marker (Kurusu et al., 2012). Rice protoplasts were isolated and transformed according to published methods (Zhang et al., 2011). The fluorescence images were captured via an inverted Zeiss LSM 780 laser scanning microscope.

### Measurement of SPAD and Agronomic Traits Analysis

Leaves of transgenic lines and SN9816 at the tillering stage were used to measure the relative chlorophyll content determined by a soil plant analysis development (SPAD) value measured using a SPAD-502 Plus chlorophyll meter (Konica Minolta, Japan). The agronomic traits including plant height, tiller number, seed-setting rate, thousand grain weight, panicle number, and grain yield were analyzed at ripening stage according to methods described previously (Bin et al., 2015). Three random pots with four replicates were chosen to be measured and the agronomic traits were all measured on a single-plant basis.

### Biomass, Total N Content, NUpE, and NUE Calculations

Rice plants were sampled at the ripening stage by separating them into culm, leaf sheath, leaf blade, and panicle at harvest, then all samples were kept in a dry oven at 80°C for 7 days, and dry masses were weighed as DW_Dr_ (dry weight of culm, leaf sheath, leaf blade, and panicle) and DW_SEEDs_ (dry weight of total seeds). Then the dry mass of these tissue organs was ground to powder and total N concentrations (N%) were measured using an Elemental Analyzer (Elementar, Vario MAX C, Germany), as N% = mg N/100 mg DW. Biomass was calculated as DW_Dr_ + DW_SEEDs_. The harvest index (HI) was calculated as DW_SEEDs_/(DW_SEEDs_+DW_Dr_) ratio and was used as an indicator of yield. The N harvest index (NHI) was calculated as (N%_SEEDs_ × DW_SEEDs_)/(N%_SEEDs_ × DW_SEEDs_ + N%_Dr_ × DW_Dr_). The NUpE was calculated as (total aboveground N accumulation in N225 or N75 pot) – (total aboveground N accumulation in N0 pot)/N supply, and the NUE was calculated as (grain yieid in N225 or N75 pot) – (grain yield in N0 pot)/N supply.

### Statistical Analysis

The experiments were repeated in three separate plots as three replicates, and the representative results were shown in the figures. The statistically significant differences of all data were analyzed based on Student’s *t*-test with the comparisons between two groups of data (transgenic lines vs. SN9816) at significance levels of *P < 0.05 and **P < 0.01.

## Results

### Subcellular Localization and Temporal and Spatial Expression Patterns of *OsATG8a* in Rice

**FIGURE 1:**
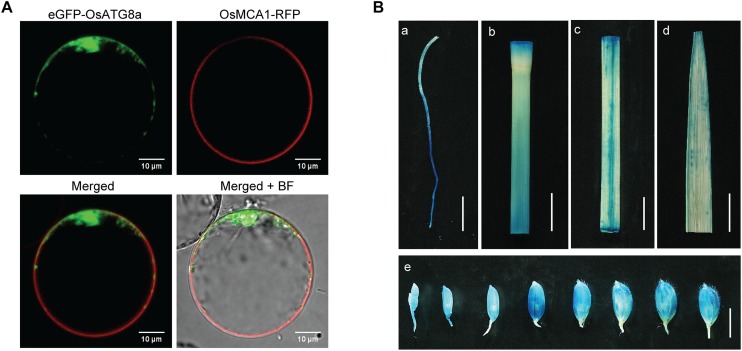
Protein subcellular localization of OsATG8a and expression patterns of *OsATG8a* gene. **(A)** Subcellular localization of OsATG8a in rice protoplasts. Panels showed the image of eGFP-OsATG8a, the cell membrane marker OsMCA1-RFP, merged of eGFP-OsATG8a and OsMCA1-RFP, and overlap image of mergerd and bright-field (BF), respectively. Scale bars, 10 μm. **(B)** GUS histochemical staining of *Pro_OsATG8a_-GUS* transgenic rice, tissues used for GUS staining including root from 14-day-old seedlings cultured hydroponically **(a)**, culm **(b)**, leaf sheath **(c)**, leaf blade **(d)**, and developing seeds **(e)**. Scale bars, 1 cm in **(a–d)**, 5 mm in **(e)**.

ATG8 is a reliable marker for autophagosomes and autophagic activity when fused with a fluorescent protein in plants and animals (Ichimura et al., 2000; Yoshimoto et al., 2004; Contento et al., 2005; Avin-Wittenberg et al., 2012; Izumi et al., 2015). Subcellular localization analysis with rice protoplasts showed that the eGFP-OsATG8a fusion protein was predominantly localized to the cytoplasm, with autophagosome-like structures seen as fluorescence puncta in rice protoplasts (Figure 1A), while the colocalization assay with a cell membrane marker OsMCA1-RFP (Kurusu et al., 2012) showed that the OsATG8a was not localized to cell membrane (not co-localized with RFP). To examine the temporal and spatial expression patterns of *OsATG8a*, we performed histochemical analysis of the promoter-GUS fusion report systems in *Pro_OsATG8a_-GUS* transgenic rice. Histochemical staining indicated that GUS activity could be detected in the root, leaf sheath, leaf blade, culm, and the developing seeds of rice (Figure 1B). When the *Pro_OsATG8a_-GUS* transgenic rice seedlings were subjected to nitrogen deficiency conditions, GUS activity significantly increased (Supplementary Figures S1A,B). To confirm whether the expression of *OsATG8a* responded to N limitation, we further examined the transcript level of *OsATG8a* using real-time RT-PCR. When rice seedlings of SN9816 were placed under N stress for 1 or 3 days, the expression levels of *OsATG8a* in leaves and roots were significantly increased with an increasing degree of N stress compared with NS conditions (Supplementary Figures S1C,D). These results suggest that expression of *OsATG8a* was induced by N stress.

### Overexpression of *OsATG8a* Increases the Number of Tillers and Reduces Plant Height in Transgenic Rice

**FIGURE 2:**
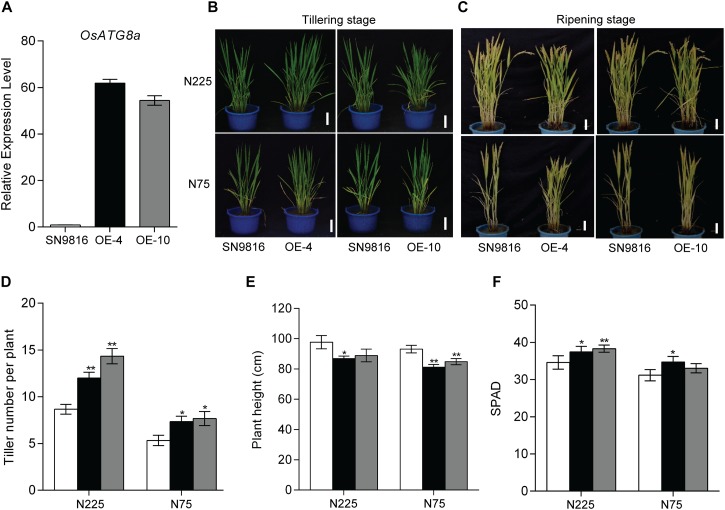
Characterization of *OsATG8a*-overexpressing transgenic rice. **(A)** Expression level of *OsATG8a* in 14-day-old seedlings of SN9816 and transgenic lines (OE-4 and OE-10). *OsActin1* was used as an internal control. Values are means ± SD (*n* = 3). Phenotypes of the whole rice plants under sufficient N (N225) or low N (N75) conditions at tillering stage **(B)** and ripening stage **(C)**. Scale bars, 10 cm. **(D)** Tiller number per plant, **(E)** plant height per plant and **(F)** SPAD value per plant of SN9816 and *OsATG8a*-overexpressing lines (OE-4 and OE-10) under N225 or N75 conditions at ripening stage. Values are means ± SD of one representative biological replicate (*n* = 12) out of three, *P < 0.05, **P < 0.01 (*t*-test).

To analyze the physiological function of *OsATG8a* in developmental processes in rice, we obtained more than 10 independent transgenic lines. The transcript level of *OsATG8a* was confirmed in two homozygous transgenic lines using real-time RT-PCR (Figure 2A). Lines 4 and 10 (OE-4, OE-10) are the over-expressed lines. Under both normal growth (N225) and low N (N75) conditions, the tiller number in the transgenic lines was significantly higher than that of the control plant (SN9816) from tillering to ripening stage and the transgenic lines were more robust and vigorous than the control (Figures 2B–D). In the ripening stage, transgenic plants were shorter than control plants (Figure 2E), especially under N75 conditions. Additionally, the relative chlorophyll content SPAD value in flag leaves of transgenic lines was higher than in the control (Figure 2F), which indicated that leaf senescence was slightly delayed in the *OsATG8a*-overexpressing rice. These results suggest that overexpression of *OsATG8a* plays a positive role in promoting rice tillering, delaying senescence, and prolonging efficient photosynthetic activities.

### Overexpression of *OsATG8a* Increases Panicle Number and Yield and Promotes NUE in Transgenic Rice

**FIGURE 3:**
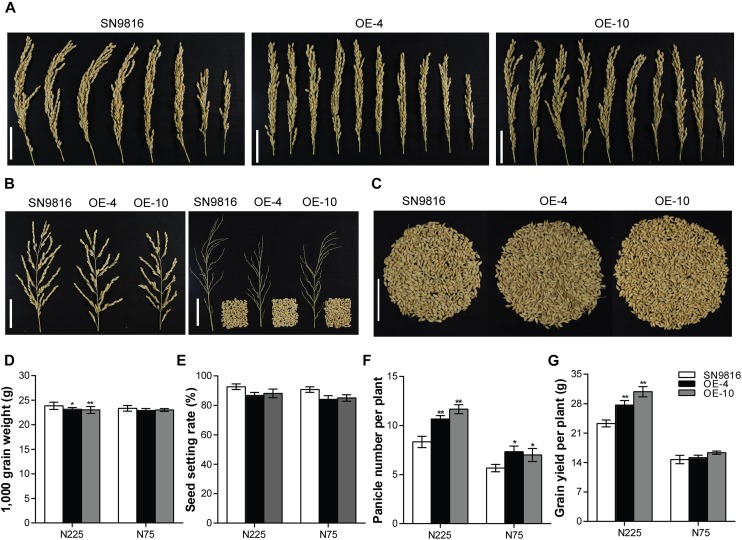
Comparison of yield-related characteristics between SN9816 and *35S-OsATG8a* transgenic rice. Phenotypes of **(A)** panicle number per plant, **(B)** panicle and grain number per panicle, and **(C)** grain number per plant in SN9816 and transgenic lines (OE-4 and OE-10) under N225 condition. Scale bars, 5 cm. **(D)** Thousand grain weight, **(E)** seed setting rate, **(F)** panicle number per plant, and **(G)** grain number per plant of SN9816 and transgenic lines (OE-4 and OE-10) under N225 or N75 conditions. Values are means ± SD of one representative biological replicate (*n* = 12) out of three, *P < 0.05, **P < 0.01 (*t*-test).

**FIGURE 4:**
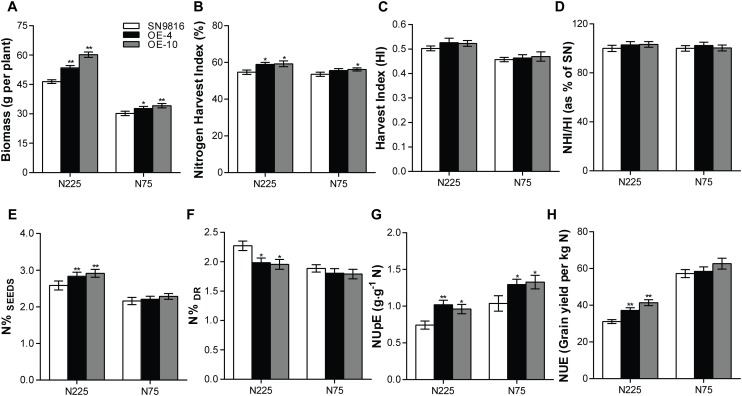
Evaluation of biomass, NUpE, and NUE in transgenic rice. **(A)** Biomass accumulation as measured by dry weight of the whole plant, **(B)** Nitrogen harvest index (NHI), **(C)** Harvest index (HI), **(D)** the ration of NHI/HI, **(E)** N concentration in seeds (N%_SEEDs_) and **(F)** in dry remains (N%_Dr_), **(G)** Nitrogen uptake efficiency (NUpE) and **(H)** nitrogen use efficiency (NUE) in SN9816 and transgenic lines (OE-4 and OE-10) under N225 or N75 conditions. Values are means ± SD of one representative biological replicate (*n* = 12) out of three, *P < 0.05, **P < 0.01 (*t*-test).

We further investigated the effect of *OsATG8a* on yield and found that the transgenic lines produced significantly more panicles and had increased yield per plant (Figures 3A,C). Although there was little difference in grain number per panicle compared to the control group, the thousand grain weight and the seed setting rate in transgenic lines were lower than those in the control plants (Figures 3B,D,E). However, the transgenic lines exhibited a significantly higher panicle number under the two N conditions (Figure 3F). These results were consistent with an increased total seed number and yield per plant compared to the control under N225, although the yield per plant was not significantly different under N75 (Figure 3G). We also measured the length and width of seeds, which showed no difference between the transgenic lines and SN9816 (Supplementary Figure S2). Given the increased yield in transgenic lines, we further analyzed plant biomass. The results showed that the biomass of transgenic lines increased by 22.29 or 10.62% under N225 or N75 conditions, respectively (Figure 4A). Harvest index (HI) and NHI are important indicators of seed production that also highlight the efficiency of N allocation to seeds. As shown in Figure 4B, the NHI was significantly higher in the transgenic lines compared to that in the control under N225 condition. The ratio of NHI to HI was used to indicate the variation of NUE as described previously (Guiboileau et al., 2012). Under both N225 and N75 conditions, the HI and the NHI/HI were not different between the transgenic lines and SN9816 (Figures 4C,D). We then analyzed the N content in seeds and dry remains, and compared to the control plant, the transgenic lines accumulated more N in seeds but had lower N in dry remains (Figures 4E,F). Since the N concentration in seeds as well as biomass were both significantly increased in transgenic lines, the NUpE and NUE were consequently increased especially under N225 condition (Figures 4G,H), suggesting that *OsATG8a* might be essential for both seed yield and NUE. Under N75 condition, the NUpE of transgenic lines was higher than that of control, but NUE was not. These results clarify that overexpression of *OsATG8a* effectively redeploys N allocation from the leaves to seeds and increases the NUE of rice under N optimal conditions.

### Overexpression of *OsATG8a* Enhances Autophagy Levels in the Transgenic Rice

**FIGURE 5:**
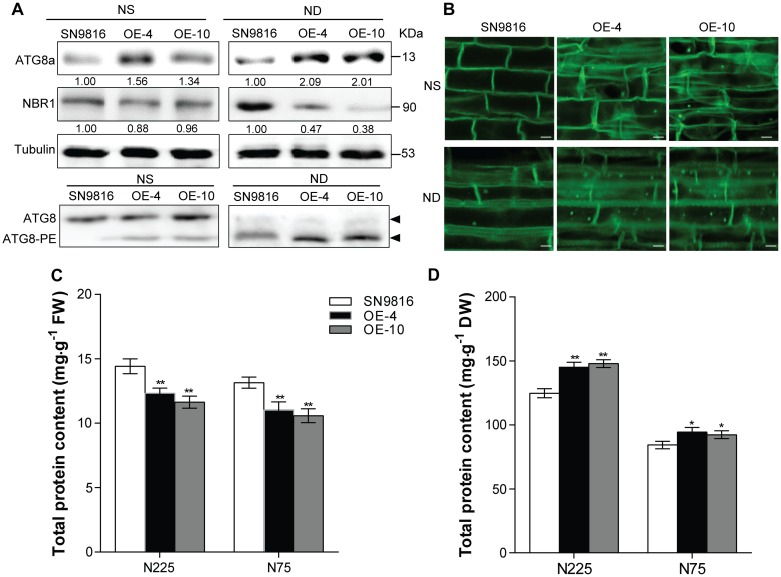
Overexpression of *OsATG8a* enhances the autophagy levels in rice. 14-day-old seedlings of SN9816 and transgenic lines (OE-4 and OE-10) cultured hydroponically in NS (3.5 mM) solution were transferred to NS (3.5 mM) or ND (0 mM) solution for 24 h (*n* = 10). **(A)** Immunoblot analysis of the extracts with anti-*Arabidopsis* ATG8a and NBR1 antibodies, near equal protein loads were confirmed by immunoblot analysis with an α-tubulin antibody. Numbers below the blot indicate intensity ratios between the NBR1 protein and the α-tubulin band in each line, and the intensity ratios in each line were normalized to SN9816 (value set to 1) under NS (3.5 mM) or ND (0 mM) conditions. The membrane fraction was used to detect the level of lipidated (ATG8-PE) and free ATG8 in the leaves. The ATG8-PE conjugates had a faster migration than ATG8 in the membrane fraction, so the band of ATG8-PE was below the band of ATG8 during SDS-PAGE in the presence of 6 M urea. **(B)** MDC-stained autophagosomes in roots were observed by confocal microscopy. Protein contents of leaves (at grain filling stage) **(C)** and seeds (after harvest) **(D)** extracts in SN9816 and transgenic lines (OE-4 and OE-10) under N225 and N75 conditions. The weight of each sample in **(C)** or **(D)** was the same, and the protein content was determined by BCA method as mg protein per gram fresh weight in leaves or mg protein per gram dry weight in seeds. Values are means ± SD (*n* = 3), *P < 0.05, **P < 0.01 (*t*-test).

The autophagy core protein ATG8a usually acts as the autophagic flux marker protein following previous reports (Klionsky and Schulman, 2014; Hafrén et al., 2017; Luo et al., 2017; Marshall and Vierstra, 2018). We first ascertained that the *OsATG8a*-overexpressing transgenic rice accumulated more ATG8a protein than control plants used ATG8a antibody of *Arabidopsis* under either NS (3.5 mM) or ND (0 mM) conditions (Figure 5A). Since the Neighbor of BRCA1 (NBR1) in *Arabidopsis* is an autophagy substrate degraded in the vacuole, the degradation level of NBR1 protein can be used to measure the selective autophagic flux in plants (Steingrim et al., 2011; Hafrén et al., 2017). Thus, we examined the autophagy receptor NBR1 by western blotting. NBR1 protein accumulation was reduced in transgenic lines compared to control plants, especially after N deficiency treatment, and thus autophagic flux increased (Figure 5A). To further analysis the autophagy flux, we monitored the lipidation of ATG8 by detected the ATG8 and lipidated ATG8 (ATG8-PE) adducts by immunoblot of the membrane protein. The ATG8-PE complex was an important component of autophagosome. The result showed that overexpression of *OsATG8a* enhanced the lipidation of ATG8, especially after N deficiency treatment, as the ATG8-PE as the level of ATG8-PE was higher in transgenic lines membranes (Figure 5A). Except that, we further used MDC staining to examine the number of autophagome in transgenic rice. MDC, an acidophilic dye widely used in mammals and plants can be used as a probe to detect autophagosome (Contento et al., 2005). There was observed more MDC-positive autophagic structures in the root cells of the transgenic lines, and the fluorescence signal intensity was higher than that in the control, especially after N deficiency treatment (Figure 5B). These results showed that overexpression of *OsATG8a* significantly increases autophagy activity and autophagic flux in transgenic rice.

Previous studies showed that autophagy is important for maintaining normal protein profiles in plants (Li F. et al., 2015; Marshall and Vierstra, 2018). To study whether overexpression of *OsATG8a* affected the protein profiles of leaves and seeds in rice, we compared the protein content in leaves or seeds of transgenic lines and control plants under N225 and N75 conditions. In leaf samples of the same quality, the protein content of leaves at the grain filling stage in transgenic lines was lower than in controls under both N conditions (Figure 5C and Supplementary Figure S3A). The protein content of seed was also higher. Corresponding to that was the protein content of seeds in transgenic lines was much higher than that of SN9816 (Figure 5D and Supplementary Figure S3B), which was consistent with the higher N content (N%_SEEDs_) in seeds of transgenic lines. These results were consistent with previous studies showing that leaf protein content in rice is affected by N stress (Wada et al., 2015), and the remobilization and reuse of N also increased due to the high autophagy level, so that most of the proteins in the leaves were transferred into the seeds, especially under N stress.

## Discussion

Autophagy appears to express at a basal level in plants under normal conditions for maintaining cell homeostasis. In plant cells, especially when nutrients are scarce, autophagy regulates the recycling and reuse of nutrients, and provides nutrients for plants by degrading and clearing macromolecules and organelles that are damaged to improve cell vitality, regulate immune response, and in adaptation to nutrient starvation. Thus, autophagy would be more important under nutrient scarcity (Bassham et al., 2006; Lenz et al., 2011; Li and Vierstra, 2012; Guiboileau et al., 2013; Michaeli et al., 2016; Hafrén et al., 2017; Minina et al., 2018). Therefore, these advantages of autophagy can be used to adjust important agricultural traits, such as increase the tolerance to abiotic and biotic stress, modulate the immune response to pathogen infection, and mitigate the effects of depleted nutrients. Nutrition limitation induces autophagy in plants (Moriyasu and Ohsumi, 1996; Thompson et al., 2005). The transcription of many plant *ATG* genes is significantly up-regulated similar to high autophagy activity under nutrient-limited conditions (Thompson et al., 2005; Chung et al., 2009; Xia et al., 2011; Wang et al., 2013). In our study, the transcript level of rice *OsATG8a* was significantly increased under N deficiency (Supplementary Figures S1C,D). Furthermore, the promoter activity of *OsATG8a* was enhanced under N-starvation conditions in the *Pro_OsATG8a_-GUS* transgenic rice (Supplementary Figures S1A,B).

We further demonstrated the role of *OsATG8a* in plant growth, yield, and nitrogen utilization. The *35S-OsATG8a* transgenic lines performed better than control SN9816 under normal growth conditions. The increased SPAD in the leaves (Figure 2F) reflected delayed leaf senescence and high nitrogen content in the “source” in transgenic rice and suggests that these plants had higher accumulation capacity of photosynthetic products during the vegetative growth stage. This in turn would provide more nutrients, especially N, for reproductive growth and yield. In this study, overexpression of *OsATG8a* significantly promoted rice tillering under both normal N and N limited conditions (Figure 2D), consistent with increased yield, especially under normal N conditions (Figure 3G). In the *OsATG8a*-overexpressing transgenic rice lines, although the number of grains per panicle did not change, the number of panicles was significantly higher than the control, which increased the yield per plant (Figures 3A,F). The increased yield was due to the increased number of effective tillers and panicles in the *35S-OsATG8a* transgenic rice. Meanwhile, the GUS activity of *OsATG8a* promoter was detected in the developing seeds (Figure 1Be), indicating that the *OsATG8a* gene may also participate in seed formation in rice. Interestingly, the *OsATG8a*-overexpressing rice was shorter (Figures 2C,E). In agronomy, shorter plant height combined with increased tiller percentage is beneficial for ideal plant type and increasing crop yield. It was shown that overexpressing the soybean *GmATG8c* gene, *AtATG5* or *AtATG7* gene in model plants *Arabidopsis* could increase seed yields and tolerance to abiotic and biotic stress (Xia et al., 2012; Minina et al., 2018). Consistent with these conclusions, it was also proved that *OsATG8a* plays a positive role in rice, meaning that it may be a feasible candidate gene for agricultural breeding improvement. In addition, using autophagy to change the nutrient cycle could produce more beneficial agronomic traits.

Autophagy plays an important role in nutrient cycling in all eukaryotes, and in plants it is especially important for remobilization and transfer of N from the leaf to the grain (Masclaux-Daubresse et al., 2017). Several *atg* deletion mutants showed phenotypes such as diminished growth and development, accelerated senescence, reduced yield, severe inhibition of growth phenotype during carbon or N deficiency, the process of N remobilization is hampered due to the lack of autophagy in leaves (Doelling et al., 2002; Thompson et al., 2005; Chung et al., 2010; Anongpat et al., 2011; Guiboileau et al., 2012, 2013; Li F. et al., 2015; Wada et al., 2015; Eguchi et al., 2017). With defective autophagy, the *atg* mutants had a defect of nitrogen remobilization, N in the cells cannot be effectively recovered or utilized, leading to the accumulation of undigested soluble proteins in their leaves. All of these could inhibit plant growth and reduce NUE of plants (Ren et al., 2014). Changing the autophagy process of crops could improve NUE and increase crop yield (Avin-Wittenberg et al., 2018). The high consumption of NBR1, increased lipidation of ATG8a (Figure 5A), and enhanced number of autophagosomes in transgenic rice especially after N deficiency treatment (Figure 5B) all suggested that the autophagy level was significantly stimulated in the *OsATG8a*-overexpressing transgenic rice. We hypothesized that the increased yield in these lines may be due to the enhanced autophagy, which promotes the remobilization of N from the leaves to seeds. Corresponding to this was that the protein content of leaves decreased while the protein content of seeds increased (Figures 5C,D), which also supports this conclusion. Compared with the control, the N concentration in dry remains was much lower in transgenic lines, but significantly higher in seeds under adequate N (Figures 4E,F), these results revealed that our transgenic rice made more efficient use of available N, transferring more N to seeds, and wasting less in non-seed areas. NHI, HI, NHI/HI and seed N concentration (N%_SEEDs_) are major indicators of NUE and seed nutritional quality (Masclaux-Daubresse et al., 2010). Overexpression of *OsATG8a* in transgenic rice resulted in higher NHI, N%_SEEDs_, and NUE (Figures 4B,E,H), indicating that *OsATG8a* plays a vital role in N allocation to seeds and NUE. Similarly, the NUpE of transgenic rice was higher under two N conditions. Under N deficiency conditions, although the NUpE was higher than the control plants in the overexpressed lines, the NUE and NHI/HI did not change, which explained why the grain yield per plant of the overexpressed lines was not significantly different from that of the control group (Figures 3G, 4D,H). At the same time, this may be the reason that N%_SEEDs_ and NHI were not significantly different (Figures 4B,E).

Constitutive expression of some *ATG8* genes in the transgenic plants not only led to better tolerance to N starvation, but also accelerated growth under optimal growth conditions (Slavikova et al., 2008; Xia et al., 2012; Li W. et al., 2015, Li et al., 2016; Kellner et al., 2016; Wang et al., 2016). Recent research from Masclaux-Daubresse’s group found that overexpression *AtATG8a*, *AtATG8e*, *AtATG8f*, or *AtATG8g* in *Arabidopsis* could not only significantly increase the number of autophagosomes, but also improved N%_SEEDs_ and at the same time reduced N loss to dry biomass under full nitrate conditions. Additionally, the N remobilization efficiency was significantly increased in *ATG8* overexpressors relative to the control line but the yield and biomass were not affected (Chen et al., 2018). Although we came to similar conclusions, in our hands, the *OsATG8a* gene powerfully affected the growth and development of rice, such that the overexpression of *OsATG8a* significantly increased effective tiller number under two N conditions, and also increased grain yield and biomass under normal growth conditions. Another point worth mentioning is that both NUE and NUpE were significantly improved in overexpressing *OsATG8a* transgenic rice than control plant.

Taken together, our results suggest that *OsATG8a* has a positive role in the maintenance of plant fitness. These results have also linked autophagy to plant nitrogen distribution/homeostasis, as well as increased yield in rice. We concluded that *OsATG8a* is an important candidate gene for promoting plant growth and development and increasing yield and NUE in rice.

## Author Contributions

FX designed the study and provided experimental materials. JY performed the GUS staining, total RNA extraction, real time RT-PCR experiments, field trials of rice, phenotypic analysis, measurement of chlorophyll and SPAD, and agronomic traits analysis. XZ performed the binary vector construction, MDC staining, protein isolation, western blot analysis and the subcellular localization assay. XL performed the rice genetic transformation and the NUpE and NUE calculations. NL performed the measurement of biomass and total N content. JY and XZ analyzed the results and prepared the figures and tables. FX and XZ wrote the manuscript. All authors discussed the results and commented on the manuscript, read and approved the final manuscript.

## Conflict of Interest Statement

The authors declare that the research was conducted in the absence of any commercial or financial relationships that could be construed as a potential conflict of interest.
