# Supplementary material for: Increased Autophagy of Rice Can Increase Yield and Nitrogen Use Efficiency (NUE)
Source: Front Plant Sci. 2019 May 7;10:584. doi: 10.3389/fpls.2019.00584 (PMC6514234; doi:10.3389/fpls.2019.00584)
Supplement: Supplementary file 1 [file Data_Sheet_1.docx]

Supplementary Material

# **Increased autophagy of rice can increase yield and nitrogen use efficiency (NUE)**

**Jinlei Yu^#^, Xiaoxi Zhen^#^, Xin Li, Nan Li, Fan Xu^*^**

**
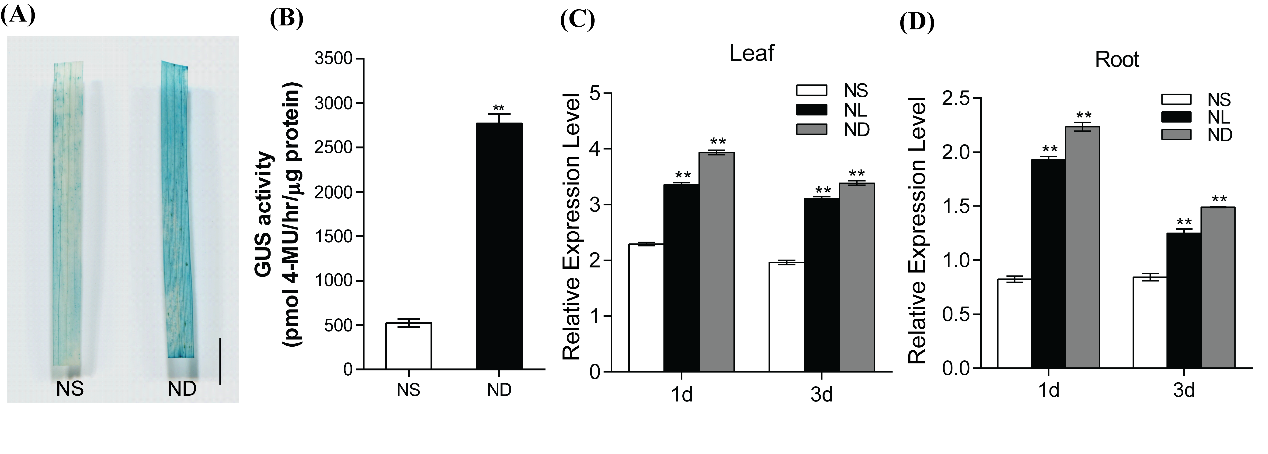
**

**Supplementary Figure 1.** The expression of *OsATG8a* was a N deficiency inducible gene of rice. GUS histochemical staining of leaves **(A)** and GUS activity (B) in *Pro_OsATG8a_-GUS* transgenic seedling treated with sufficient N (NS, 3.5 mM) or deficient N (ND, 0 mM) solution for 24 h (n=20). Scale bars, 5 mm. The rice seedlings cultured with NS solution for 14 days, and transferred to the same NS solution as the control, low N (NL) solution and the deficient N (ND) solution for 1 d and 3 d, respectively. The expression of *OsATG8a* gene in leaves **(C)** and the expression of *OsATG8a* gene in roots **(D)** were detected by real-time RT-PCR. *OsActin1* was used as an internal control. Values are means ± SD of one representative biological replicate (n = 10) out of three, **P* < 0.05, ***P* < 0.01 (t-test).

**
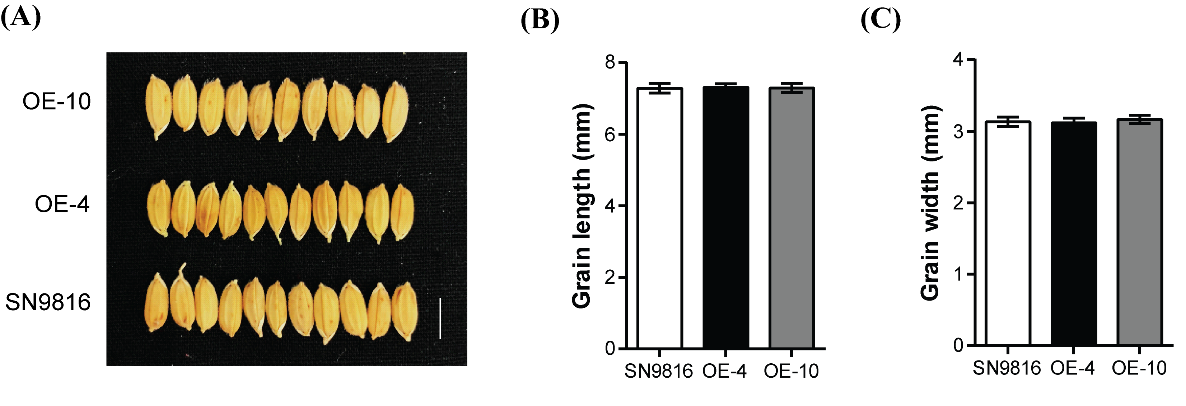
Supplementary Figure 2.** There was no change in seed size between SN9816 and the transgenic lines. The image **(A)**, the length **(B)** and width **(C)** of seeds in SN9816 and the transgenic lines (OE-4 and OE-10). Scale bars, 5 mm. Values are means ± SD of one representative biological replicate (n = 30) out of three, **P* < 0.05, ***P* < 0.01 (t-test).


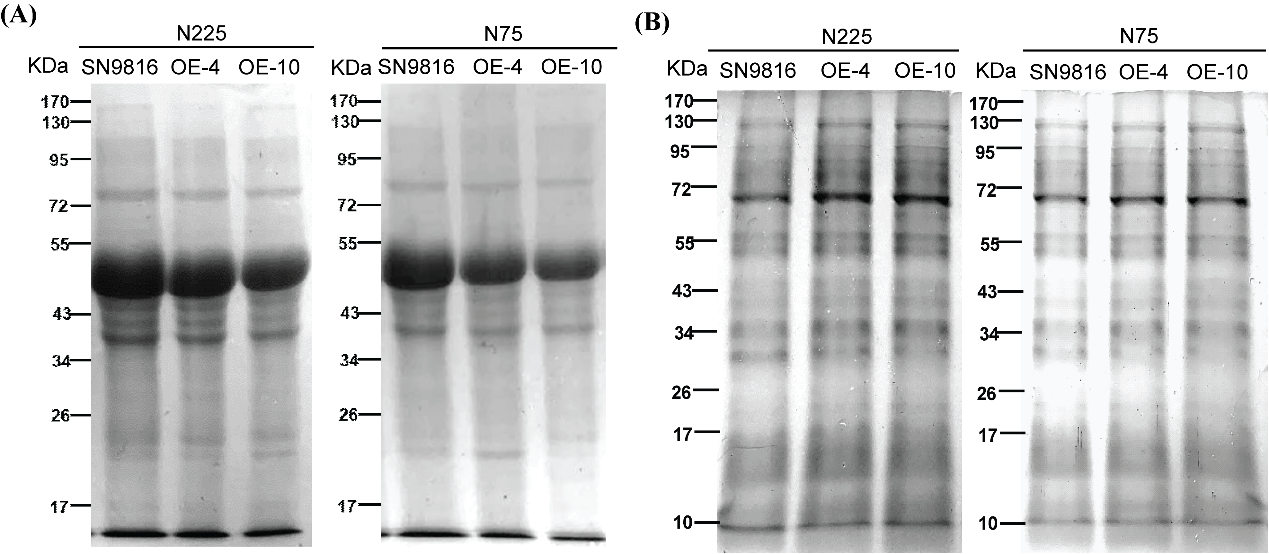
**Supplementary Figure 3.** Protein profiles of leaves (at grain filling stage) **(A)** and seeds (after harvest) **(B)** extracts in SN9816 and transgenic lines (OE-4 and OE-10) under N225 and N75 conditions separated by SDS-PAGE and stained for protein with Coomassie Brilliant Blue. Proteins were extracted from the same weight of leaves (100mg FW in 200ul buffer) or seeds (5mg of DW in 200ul buffer). The same volume was separated by 12% SDS–PAGE.

**Supplementary Table 1** The information of primers used in this study.

| Primer name | Sequence (5´→ 3´) | Functions |
| --- | --- | --- |
| cOsATG8a-F | CCATGGATGGCCAGGACTTCCTTCAAGCT | Gene cloning |
| cOsATG8a-R | GGTGACCTTACGCAGAGCCGAATGTGTTCTC | Gene cloning |
| cpOsATG8a-F | GAATTCCGTCCAAACAATTCCCTTATCTAAAAGAA | Gene cloning |
| cpOsATG8a-R | CCATGGCTCCAACCTGCGAATCAAATCAT | Gene cloning |
| rtOsATG8a-F | ACTGGAAAGGAGGCAAGCAG | Real-time RT-PCR |
| rtOsATG8a-R | CCTTCTCAACGATCACCGGA | Real-time RT-PCR |
| rtOsActin1-F | ACCATTGGTGCTGAGCGTTT | Real-time RT-PCR |
| rtOsActin1-R | CGCAGCTTCCATTCCTATGAA | Real-time RT-PCR |
